# Diagnosis and Subtyping of Autoimmune Encephalitis Using an Attention‐Based Multi‐Instance Learning Model: A Multi‐Center ^18^F‐FDG PET Study

**DOI:** 10.1111/cns.70513

**Published:** 2025-08-04

**Authors:** Yueqian Sun, Ruizhe Sun, Jiahua Lv, Qingxia Kong, Cixiang Dai, Bin Wang, Xiong Han, Min Chen, Ruihan Liu, Yan Jiang, Leilei Yuan, Lin Ai, Xiaodong Yang, Yiqiang Chen, Qun Wang

**Affiliations:** ^1^ Department of Neurology, Beijing Tiantan Hospital Capital Medical University Beijing China; ^2^ Institute of Computing Technology Chinese Academy of Sciences Beijing China; ^3^ Clinical Medical College Jining Medical University Jining China; ^4^ Department of Neurology Affiliated Hospital of Jining Medical University Jining China; ^5^ Department of Neurology Hebei Yanda Hospital Sanhe China; ^6^ Department of Neurology People's Hospital of Henan Zhengzhou China; ^7^ Department of Neurology The First Affiliated Hospital of Zhengzhou University Zhengzhou China; ^8^ Department of Neurology Xiangya Hospital of Central South University Changsha China; ^9^ Department of Nuclear Medicine, Beijing Tiantan Hospital Capital Medical University Beijing China; ^10^ National Center for Clinical Medicine of Neurological Diseases Beijing China

**Keywords:** ^18^F‐FDG PET, attention‐based, autoimmune encephalitis, multi‐instance learning

## Abstract

**Background:**

The aim was to develop an attention‐based model using ^18^F‐fluorodeoxyglucose (^18^F‐FDG) PET imaging to differentiate autoimmune encephalitis (AE) patients from controls and to discriminate among different AE subtypes.

**Methods:**

This multi‐center retrospective study enrolled 390 participants: 222 definite AE patients (comprising four subtypes: LGI1‐AE, NMDAR‐AE, GABAB‐AE, GAD65‐AE), 122 age‐ and sex‐matched healthy controls, and 33 age‐ and sex‐matched antibody‐negative AE patients along with 13 age‐ and sex‐matched viral encephalitis patients, both serving as disease controls. An attention‐based multi‐instance learning (MIL) model was trained using data from one hospital and underwent external validation with data from other institutions. Additionally, a multi‐modal MIL (m‐MIL) model integrating imaging features, age, and sex parameters was evaluated alongside logistic regression (LR) and random forest (RF) models for comparative analysis.

**Results:**

The attention‐based m‐MIL model outperformed classical algorithms (LR, RF) and single‐modal MIL in AE vs. all controls binary classification, achieving the highest accuracy (84.00% internal, 67.38% external) and sensitivity (90.91% internal, 71.19% external). For multiclass AE subtype classification, the MIL‐based model achieved 95.05% (internal) and 77.97% (external) accuracy. Heatmap analysis revealed that NMDAR‐AE involved broader brain regions, including the medial temporal lobe (MTL) and basal ganglia (BG), whereas LGI1‐AE and GABAB‐AE showed focal attention on the MTL and BG. In contrast, GAD65‐AE demonstrated concentrated attention exclusively in the MTL.

**Conclusion:**

The m‐MIL model effectively discriminates AE patients from controls and enables subtyping of different AE subtypes, offering a valuable diagnostic tool for the clinical assessment and classification of AE.

## Graphical abstract

This study presents an attention‐based model for distinguishing autoimmune encephalitis (AE) from controls and subtyping AE using ^18^F‐FDG PET. Analyzing 390 participants (222 AE, 122 healthy controls (HC), 46 disease controls (DC)), the model showed high accuracy in AE‐control discrimination and identified subtype‐specific brain metabolic patterns. The m‐MIL framework integrates PET features (via ResNet18 extraction from 79 brain slices), clinical data (One‐Hot encoded sex/age), and attention mechanisms for binary classification, with high‐weight layers enabling AE subtyping.

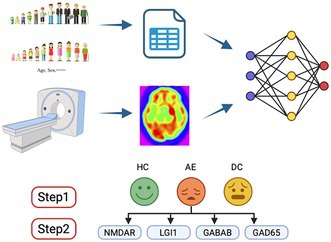

**Abbreviations**
^18^F‐FDG PET: ^18^F‐fluorodeoxyglucose positron emission tomography
ACC: accuracy
AE: autoimmune encephalitis
AUC: area under the curve
BG: basal ganglia
CASPR2: contactin‐associated protein‐like 2
CBAs: cell‐based assays
CSF: cerebrospinal fluid
DCs: disease controls
EEG: electroencephalography
GABABR: gamma‐aminobutyric acid B receptor
GAD65: glutamic acid decarboxylase 65
HCs: healthy controls
LGI1: leucine‐rich glioma‐inactivated 1 protein
LR: logistic regression
MIL: multi‐instance learning
MNI: Montreal Neurological Institute
MTL: medial temporal lobe
NMDAR: N‐methyl‐D‐aspartate receptor
RF: random forest
SEN: sensitivity
SPE: specificity
SPM12: statistical parametric mapping 12
VE: viral encephalitis

## 1 Introduction

Autoimmune encephalitis (AE) is an immune‐mediated disorder where autoantibodies recognize and damage neuronal synapses and cell surfaces [1, 2]. Since the 2007 discovery of AE characterized by anti‐N‐methyl‐D‐aspartate receptor (NMDAR) antibodies, numerous encephalitis‐associated autoantibodies against neuronal components have been identified. These include antibodies against leucine‐rich glioma‐inactivated 1 protein (LGI1), contactin‐associated protein‐like 2 (CASPR2), gamma‐aminobutyric acid B receptor (GABABR), and glutamic acid decarboxylase 65 (GAD65), among others. AE incidence has tripled in recent decades, largely due to expanded clinical diagnostic criteria and improved autoantibody detection methods, making its prevalence comparable to that of infectious encephalitis [3, 4].

Early detection of AE significantly improves clinical outcomes, and prognoses vary across its subtypes [5], yet current diagnosis relies on time‐consuming autoantibody testing in serum and cerebrospinal fluid (CSF) [4, 6], with specific antibodies enabling subtype identification. A significant subset of patients—labeled “antibody‐negative AE”—exhibit clinical features of AE but lack detectable neural autoantibodies, necessitating differentiation from definite AE cases. The absence of autoantibodies in these cases hinders prognosis prediction and immunotherapy optimization, highlighting an unmet clinical need [6, 7, 8].

Brain MRI has been proposed as a pre‐antibody diagnostic tool, but its sensitivity is limited to cases with neuronal damage or cortical atrophy [4]. Compounding the challenge, differentiating AE from viral encephalitis (VE)—which shares clinical hallmarks like cognitive‐behavioral abnormalities and seizures—remains difficult due to overlapping symptoms, CSF profiles, and imaging findings [9, 10]. VE, often caused by herpes simplex or enteroviruses, exemplifies this diagnostic ambiguity, underscoring the need for robust discriminative markers.

^18^F‐fluorodeoxyglucose positron emission tomography (^18^F‐FDG PET) has emerged as a cornerstone for neuroimmune disease diagnosis, enabling noninvasive visualization of metabolic aberrations that precede structural changes in AE [11]. Clinically, it is recommended when MRI is normal or contraindicated, with studies showing superior sensitivity for AE‐related abnormalities—significantly higher than MRI [4, 5, 12]. Notably, FDG‐PET detects aberrant brain metabolism weeks earlier than electroencephalography (EEG), MRI, or CSF changes [13], while subtype‐specific metabolic signatures suggest its potential as a diagnostic discriminator.

In clinical practice, imaging results have traditionally been interpreted visually to facilitate patient diagnoses [14], but this subjective assessment of PET data can introduce variability. To address this, there is a growing trend toward objective analytical methods that enhance diagnostic speed and precision [15, 16, 17]. Multi‐instance learning (MIL), a weakly supervised machine learning approach [18], has gained traction in medical imaging [19], with applications in Alzheimer's disease [20], cerebral small vessel disease [21], and other conditions. Unlike traditional image recognition requiring exact labels for every image, MIL is well‐suited for datasets with partial labels—such as patient‐level diagnoses without slice‐by‐slice annotations [22, 23, 24, 25, 26]. By integrating an attention mechanism, the model prioritizes slices with pathological changes while excluding normal regions, reducing training noise and simplifying the process. This improves model efficacy, enabling more accurate diagnoses to assist neuroscientists in patient evaluation. This study aims to develop an attention‐based MIL model to differentiate AE patients from controls and classify AE subtypes.

## 2 Materials and Methods

### 2.1 Study Design

This multi‐center retrospective study included patients from Beijing Tiantan Hospital, Henan Provincial People's Hospital, Hebei Yanda Hospital, and the Affiliated Hospital of Jining Medical University between February 2018 and July 2024. Local ethics committees at each institution approved the study.

### 2.2 Patient Selection

The inclusion criteria were as follows: aged 6–85 years; positive antibodies in both serum and CSF via cell‐based assays (CBAs; Euroimmun, Lubeck, Germany); clinical symptoms (seizures, behavioral/mood disturbances, cognitive dysfunction); and available ^18^F‐FDG PET scans. The exclusion criteria were: seizures within 2 h before PET; motion artifacts or insufficient glucose uptake; seizures due to structural brain lesions (tumors, strokes, traumatic injuries); or seizures related to comorbidities (malignant hypertension, renal/hepatic failure, severe hypoglycemia/hyperglycemia).

### 2.3 Control Groups

Healthy controls (HCs): 122 age‐/sex‐matched individuals without neuropsychiatric history (brain injury, dementia, ataxia, multiple sclerosis, epilepsy, tumors, stroke, or parkinsonism) were recruited from the same institutions. Disease controls (DCs): 33 age‐/sex‐matched antibody‐negative AE patients and 13 VE patients from Beijing Tiantan Hospital were enrolled.

### 2.4 Diagnostic Criteria

Definite AE was diagnosed per Graus et al. [4] criteria, requiring: (1) Subacute onset (< 3 months) of working memory deficits (short‐term loss), altered mental status, or psychiatric symptoms. (2) At least one of: new focal CNS findings; unexplained seizures; CSF pleocytosis (WBC > 5/mm^3^); MRI suggestive of encephalitis. (3) Exclusion of alternative causes. (4) Laboratory confirmation of neuronal cell‐surface or synaptic protein antibodies.

Antibody‐negative AE criteria included: (1) Subacute cognitive/mental symptoms. (2) At least 2 of: MRI features consistent with AE; CSF pleocytosis; CSF oligoclonal bands/elevated IgG index; brain biopsy showing inflammation.

Confirmed VE followed 2013 IEC criteria [27]: (1) Altered mental status (consciousness/lethargy/personality change) ≥ 24 h, no alternative cause. (2) At least 3 of: Fever ≥ 38°C within 72 h of presentation. Unexplained seizures. New focal neurologic deficits. CSF WBC ≥ 5/mm^3^. Neuroimaging showing acute brain parenchyma abnormalities (new or acute‐onset). EEG abnormalities consistent with encephalitis (non‐attributable to other causes). (3) Viral infection confirmed by PCR/serology.

### 2.5 PET Imaging and Preprocessing

PET data were processed using MATLAB 2019b and SPM12 [28]. Images were spatially normalized to MNI space (12‐parameter affine/nonlinear transformations, 2 × 2 × 2 mm voxels) and smoothed with an 8‐mm Gaussian kernel to enhance signal‐to‐noise ratio.

### 2.6 Feature Extraction and Fusion

**FIGURE 1:**
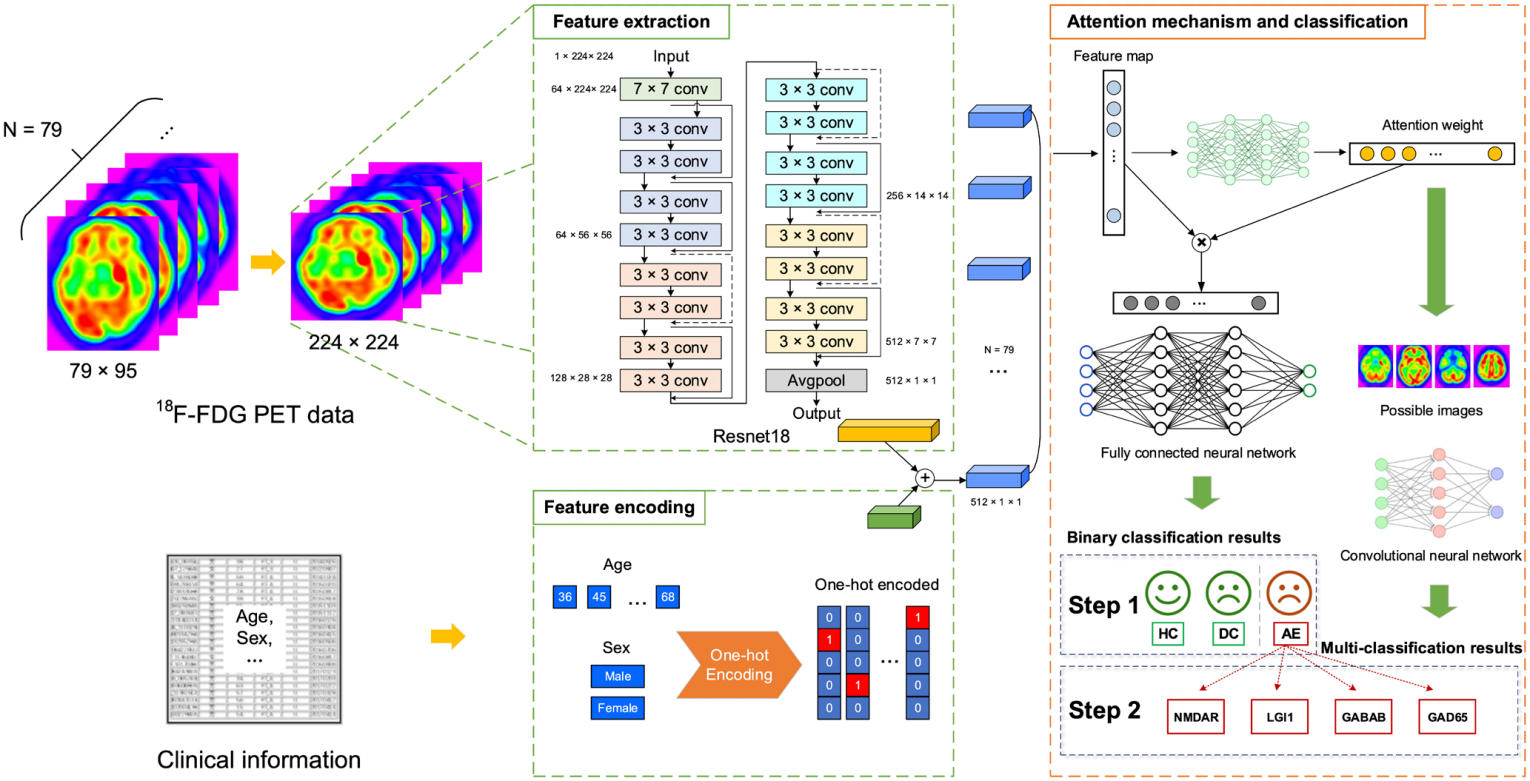
Framework of the proposed multi‐instance learning model. For PET imaging data, 79‐layer cross‐sectional images for each patient were transformed and the resnet18 network was used for feature extraction, yielding a 512‐dimensional block. One‐Hot encoding was used to represent discrete clinical information including sex, forming a feature block. Concatenation was used to connect the two, which were then inputted into a neural network based on an attention mechanism. The developed network initially performed the binary classification of AE and control samples. A layer with higher attention weights was then used for multi‐classification to distinguish among AE subtypes. AE, autoimmune encephalitis; DCs, disease controls; HCs: healthy controls.

The 18‐layer ResNet18 convolutional neural network [29] was utilized to extract imaging features. Original PET axial images (79 × 95 pixels) were resized to 224 × 224 pixels. Modifying the average pooling layer in the original network enhanced the model's focus on local feature abnormalities. By excluding the fully connected layer to directly output feature information rather than classification results, a 512‐dimensional image feature sequence was generated. Clinical data (age and sex) were digitized via One‐Hot Encoding and fused with image features. A neural network addressed model imbalance through feature compression, followed by fusion and expansion to 512 dimensions (Figure 1).

### 2.7 Multi‐Instance Learning and Attention Modeling

MIL classified PET slices by treating each patient's 79 slices as a “bag” (AE bags positive, HC/DC bags negative). Positive bags contained lesion‐positive/negative instances; negative bags had all negative instances. Beijing Tiantan Hospital data were split into training (70%) and internal validation (30%) cohorts. An attention mechanism26 assigned weights via:

$$s=f\left(a_{i},h_{i}\right)$$

$$a_{i}=\frac{\exp\left(w^{T}\tanh\left(h_{i}^{T}\right)\right)}{\sum_{j=1}^{K}\exp\left(w^{T}\tanh\left(h_{i}^{T}\right)\right)}$$

where *f* is a dot product alignment function, *a*_i_ is the attention weight, $h_{i}$ denotes the *i*th instance feature embedding in the bag, and *w* ^T^ is a neural network–determined linear transformation. The multi‐modal MIL (m‐MIL) model integrated imaging, age, and sex parameters, validated against single‐modal models, logistic regression (LR), and random forest (RF), with external validation using three other hospitals' data.

### 2.8 Statistical Analysis

Python (v 3.8.5) was used for statistical analyses, with data that were normally distributed being reported as means ± standard deviation (SD). Model performance was evaluated using the area under the curve (AUC), accuracy (ACC), sensitivity (SEN), specificity (SPE), F1 score, and precision.

## 3 Results

### 3.1 Patient Demographics

**FIGURE 2:**
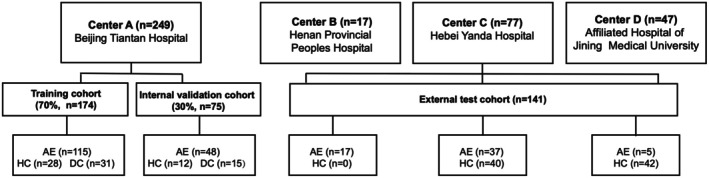
Patient distributions in the AE and control cohorts. AE, autoimmune encephalitis; DCs, disease controls; HCs, healthy controls.

**FIGURE 3:**
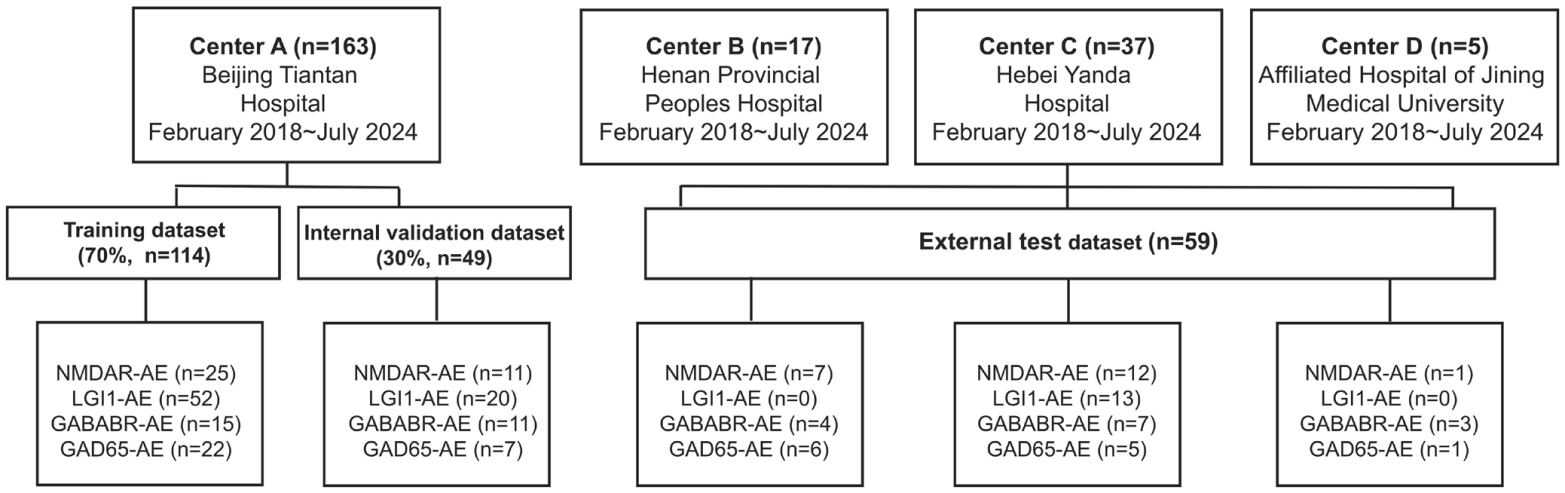
Participant distributions among AE subtypes. AE, autoimmune encephalitis; GABAB‐AE, Gamma‐aminobutyric acid type B receptor encephalitis; GAD65‐AE, glutamic acid decarboxylase 65 encephalitis; LGI1‐AE, Leucine‐rich glioma‐inactivated protein 1 encephalitis; NMDAR‐AE, N‐methyl‐D‐aspartate receptor encephalitis.

This study enrolled 222 confirmed AE patients, 122 HCs, and 46 DCs from multiple centers. Of the enrolled AE patients, 85, 56, 40, and 41 had LGI1‐AE, NMDAR‐AE, GABAB‐AE, and GAD65‐AE, respectively (Tables 1 and 2, Figures 2 and 3).

**TABLE 1:** Demographic information of subjects across different cohorts for distinguishing AE from controls.

Characteristic	Center A (Beijing Tiantan Hospital) (*n* = 249)			External test cohort (*n* = 141)
	Training cohort (70%, *n* = 174)	Internal validation cohort (30%, *n* = 75)	All	
AE				
Age, years	48.74 ± 16.34	47.96 ± 16.06	48.51 ± 16.21	41.93 ± 19.00
Gender	67/48	30/18	97/66	30/29
HCs				
Age, years	49.11 ± 10.81	50.17 ± 8.35	49.43 ± 10.04	49.17 ± 15.43
Gender	16/12	9/3	25/15	41/41
DCs				
Age, years	41.23 ± 18.20	46.53 ± 21.64	42.96 ± 19.31	NA
Gender	15/16	6/9	21/25	NA

*Note:* Categorical variables were recorded as *n* (%). The gender is reported as male/female. The age is reported as mean ± standard deviation.

Abbreviations: AE, autoimmune encephalitis; DCs, disease controls; HCs, healthy controls; VE, viral encephalitis.

**TABLE 2:** Demographic information of subjects across different datasets for differentiating among various subtypes of AE.

Characteristic	Center A (Beijing Tiantan Hospital) (*n* = 163)			External test cohort (*n* = 59)
	Training Dataset (70%, *n* = 114)	Internal validation Dataset (30%, *n* = 49)	All	
Age, y	48.59 ± 16.87	48.33 ± 14.74	48.51 ± 17.39	41.93 ± 19.00
Gender	70/44	27/22	97/66	30/29
Subtypes (*n*, %)				
NMDAR‐AE	25 (21.93%)	11 (22.45%)	36 (22.09%)	20 (33.90%)
LGI1‐AE	52 (45.61%)	20 (40.82%)	72 (44.17%)	13 (22.03%)
GABAB‐AE	15 (13.16%)	11 (22.45%)	26 (15.95%)	14 (23.73%)
GAD65‐AE	22 (19.30%)	7 (14.29%)	29 (17.79%)	12 (20.34%)

*Note:* Categorical variables were recorded as *n* (%). The gender is reported as male/female. The age is reported as Mean ± Standard deviation.

Abbreviations: AE, autoimmune encephalitis; GABAB‐AE; gamma‐aminobutyric acid type B receptor encephalitis; GAD65‐AE; glutamic acid decarboxylase 65 encephalitis; HCs; healthy controls; LGI1‐AE; leucine‐rich glioma‐inactivated protein 1 encephalitis; NMDAR‐AE; N‐methyl‐D‐aspartate receptor encephalitis.

### 3.2 Model Performance

**FIGURE 4:**
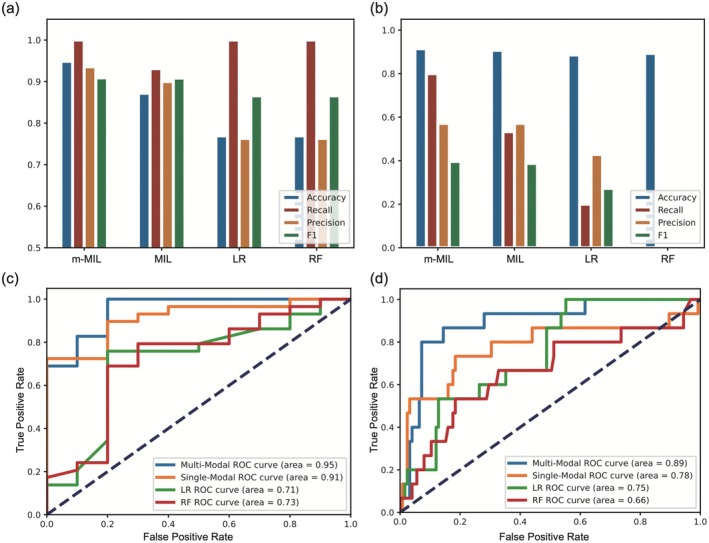
Performance of the established model for differentiating AE patients from HCs. The model was trained on a cohort from Center A and evaluated using (a) an internal validation cohort from Center A and (b) external validation cohorts from Centers B, C, and D. Receiver operating characteristic (ROC) curves for (c) internal and (d) external validation datasets show the area under the curve (AUC) for tested models. AE, autoimmune encephalitis; HCs, healthy controls.

In the binary classification of AE versus HCs, the m‐MIL model outperformed MIL and classical algorithms (LR, RF) in both internal and external validation (Figure 4, Table 3). It achieved the highest accuracy (94.87% internally, 91.43% externally) and sensitivity (100% internally, 80% externally), while traditional models showed poor generalizability: LR and RF had low sensitivity (20.00%, 0.00%) and precision (42.86%, 0.00%), and MIL exhibited reduced diagnostic efficiency externally. For specificity, m‐MIL, MIL, LR, and RF showed 0.80, 0.70, 0.10, and 0.10 in internal validation and 0.94, 0.95, 0.97, and 1.00 in external validation. The m‐MIL also had the highest AUC in internal validation, underscoring its robustness for clinical AE diagnosis.

**TABLE 3:** Performance comparison of m‐MIL, MIL, LR, and RF models in internal and external validation for binary classification of AE versus HC.

Model	Validation	ACC (%)	SEN (%)	PRE (%)	F1 (%)
m‐MIL	Internal	94.87	100.00	93.55	90.89
	External	91.43	80.00	57.14	39.63
MIL	Internal	87.18	93.10	90.00	90.84
	External	90.71	53.33	57.14	38.73
LR	Internal	76.92	100.00	76.32	86.57
	External	88.57	20.00	42.86	27.27
RF	Internal	76.92	100.00	76.32	86.57
	External	89.29	0.00	0.00	0.00

*Note:* Categorical variables were recorded as *n* (%).

Abbreviations: ACC, accuracy; AE, autoimmune encephalitis; F1, F1‐score; HC, healthy control; LR, logistic regression; m‐MIL, multi‐modal multi‐instance learning; MIL, multi‐instance learning; PRE, precision; RF, random forest; SEN, sensitivity.

**FIGURE 5:**
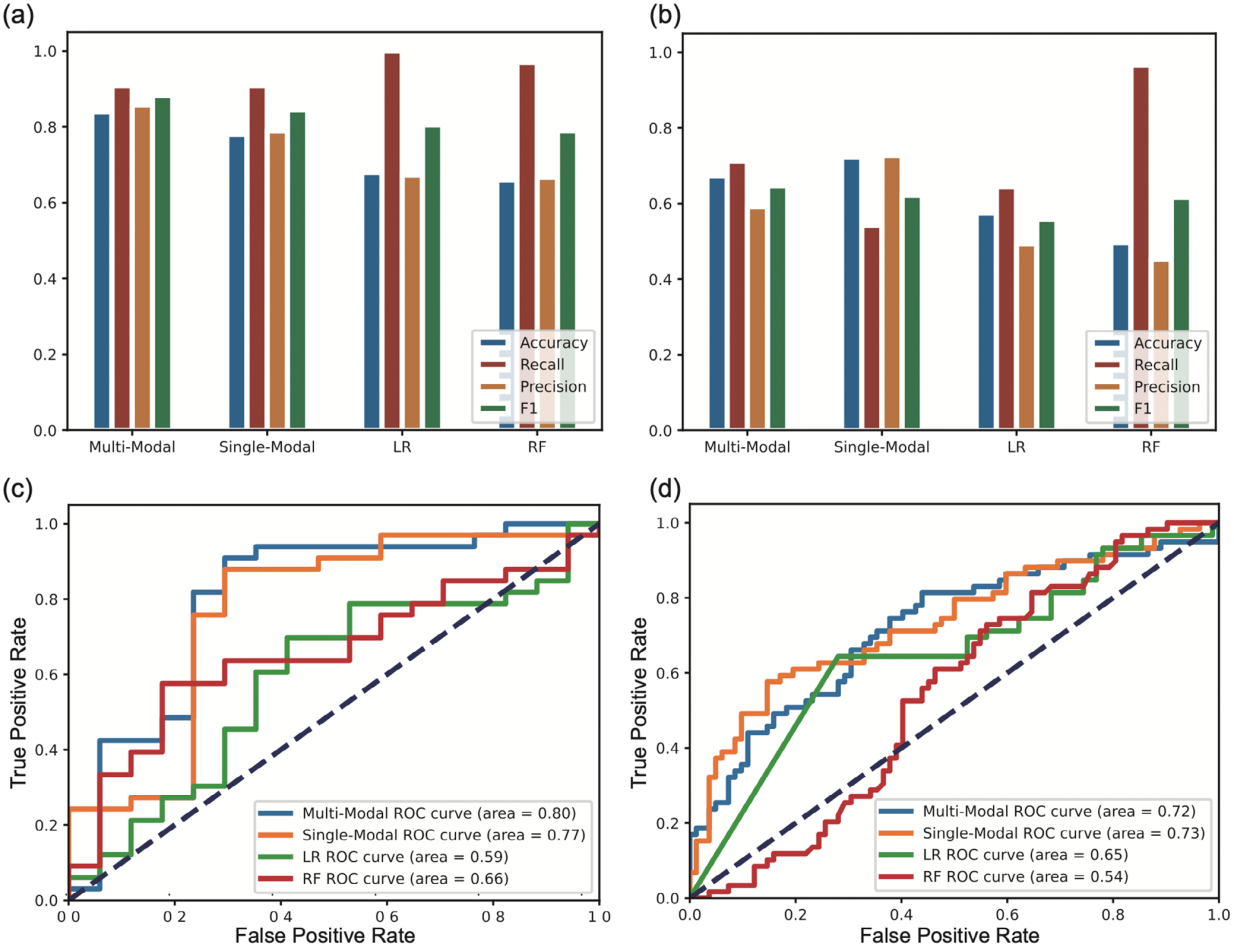
Performance of the established model for differentiating AE patients from all controls. The model was trained on a cohort from Center A and evaluated using (a) an internal validation cohort from Center A and (b) external validation cohorts from Centers B, C, and D. Receiver operating characteristic (ROC) curves for (c) internal and (d) external validation datasets show the area under the curve (AUC) for tested models. AE, autoimmune encephalitis.

For distinguishing AE from all controls, model performances varied: m‐MIL and MIL both showed consistent stability across validations, while LR and RF exhibited divergent performances, reflecting model‐specific generalization differences. Internal and external validation results are presented in Figure 5 and Table 4. In terms of specificity, m‐MIL, MIL, LR, and RF exhibited 0.70, 0.53, 0.06, and 0.06 in internal validation and 0.65, 0.85, 0.53, and 0.16 in external validation.

**TABLE 4:** Performance comparison of m‐MIL, MIL, LR, and RF models in internal and external validation for binary classification of AE versus controls.

Model	Validation	ACC (%)	SEN (%)	PRE (%)	F1 (%)
m‐MIL	Internal	84.00	90.91	85.71	88.24
	External	67.38	71.19	59.15	64.62
MIL	Internal	78.67	90.91	78.95	84.51
	External	72.34	54.24	72.73	62.14
LR	Internal	68.00	100.00	67.35	80.49
	External	57.45	64.41	49.35	55.88
RF	Internal	66.67	96.97	66.67	79.01
	External	49.65	96.61	45.24	61.62

*Note:* Categorical variables were recorded as *n* (%).

Abbreviations: ACC, accuracy; AE, autoimmune encephalitis; F1, F1‐score; LR, logistic regression; m‐MIL, multi‐modal multi‐instance learning; MIL, multi‐instance learning; PRE, precision; RF, random forest; SEN, sensitivity.

For the multiclass classification of patients into AE subtypes, the MIL‐based model achieved an accuracy rate of 95.05% in internal validation, with external validation yielding 77.97% accuracy. As the other classical models lacked accurate image‐level training data, they could not be used to conduct these multiclass classification analyses.

**FIGURE 6:**
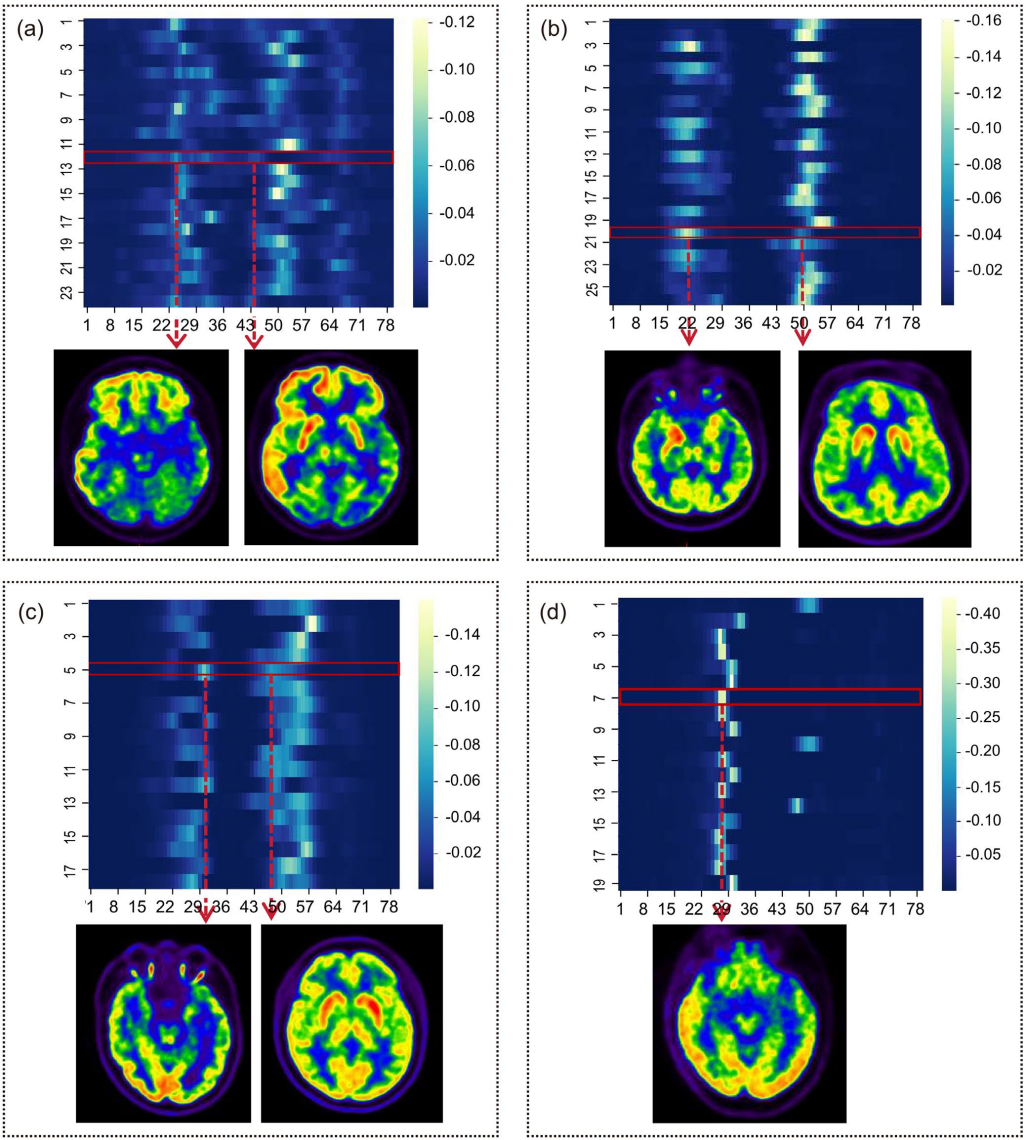
Heatmap overview of differences in PET images among patients from the Tiantan Hospital training cohort. The horizontal and vertical axes respectively denote patients and PET slices. Brightness is proportional to attention weights, with the values for each row summing to 1. (a) NMDAR‐AE: Patient #12 (20‐year‐old female); the red box highlights two brighter regions exhibiting hypometabolism in the temporal lobes and hypermetabolism in basal ganglia. (b) LGI1‐AE: Patient #20 (65‐year‐old female); the red box highlights an area of hypermetabolism in the bilateral temporal lobes, hippocampi, amygdalae, and basal ganglia. (c) GABAB‐AE: Patient #5 (48‐year‐old male); the red box highlights an area of hypometabolism in the bilateral hippocampi, amygdalae, and hypermetabolism in the basal ganglia. (d) GAD65‐AE: Patient #7 (20‐year‐old female); the red box highlights a bright region indicative of hypometabolism in the right temporal lobe.

In a heatmap with patients aligned along the horizontal axis and PET image slices along the vertical axis, distinct PET image patterns were observed for each patient in the Beijing Tiantan Hospital training cohort. This visualization depicts attention weights assigned to individual slices, where higher weights correspond to brighter intensities—highlighting the model's focus on diagnostically significant slices. Each row's weights summed to 1, illustrating the attention distribution across slices. For most AE patients, slices containing the medial temporal lobe (MTL) showed the greatest differentiation from HCs (Figure 6).

Notably, NMDAR‐AE patients exhibited more extensive slice involvement than other subtypes: attention weights were high not only in MTL slices but also in those covering the basal ganglia (BG). LGI1‐AE and GABAB‐AE patients showed elevated weights in MTL and BG slices, whereas GAD65‐AE patients demonstrated concentrated attention solely on MTL‐containing slices.

## 4 Discussion

In this study, an attention‐based m‐MIL model was developed to discriminate between AE patients and controls, as well as among AE subtypes. This model was also compared to the MIL, RF, and LR models, revealing its superior performance. These findings highlight the m‐MIL model's potential as a robust tool for AE diagnosis and subtyping.

While elevated ^18^F‐FDG uptake reflects AE‐related inflammation, nonspecific metabolic patterns may overlap with other pathologies. Moreover, this metabolic dysregulation is not evident in a subset of AE patients [30]. Diagnosing AE early in its course remains challenging for patients exhibiting nonspecific symptoms, and autoantibody detection is often delayed or unavailable. Current diagnostic criteria only include ^18^F‐FDG PET for definite autoimmune limbic encephalitis, potentially due to limited data on its predictive value for specific AE subtypes [31]. This underscores the need for advanced diagnostic approaches.

The m‐MIL model developed herein outperformed other tested models for all tested parameters, and exhibited particularly good sensitivity. As conventional LR‐ and RF‐based approaches are limited by difficulties locating the layers containing lesions, the resultant trained models tend to exhibit poor performance. While the accuracy of the developed model declined somewhat during external validation, likely owing to differences in test and training data distributions, it continued to outperform all other tested models. Notably, the m‐MIL model exhibited superior discriminative ability for definite AE against disease controls, including antibody‐negative AE and VE. This highlights the model's robustness and underscores the critical role of ^18^F‐FDG PET metabolic patterns in diagnosing and differentiating definite AE from other etiologies.

In prior studies, researchers have sought to leverage PET images to facilitate the differentiation among subtypes of AE, revealing that AE patients present with distinctive ^18^F‐FDG PET patterns, which include hypermetabolic activity in the medial temporal and striatal areas together with diffuse cortical hypometabolism [13, 32, 33]. In the present study, the majority of the image slices that presented with relatively clear differences between the AE and HC groups were those containing the MTL and BG.

Early stages of NMDAR‐AE typically exhibit hypometabolism in the occipital and other posterior brain regions [34, 35]. Diverse hypermetabolic patterns, extending beyond the BG and MTL to involve additional cortical areas, have been observed [36]. Consistently, our model prioritized image slices covering a broader regional scope for NMDAR‐AE classification, specifically including the MTL and occipital lobes. Notably, widespread hypometabolism was evident in the cerebellum and other cortical areas, aligning with the clinical heterogeneity of NMDAR‐AE. Larger sample sizes in future studies may enable more objective analytical approaches. LGI1‐AE is the second most common AE subtype after NMDAR‐AE [37]. Western blot analyses of patient brain samples have revealed LGI1 protein expression in the pallidus, hippocampus, and BG [38]. Wegner et al. [39] characterized metabolic patterns distinguishing LGI1‐AE from NMDAR‐AE, identifying BG hypermetabolism as a hallmark of LGI1‐AE. Correspondingly, our m‐MIL model prioritized image slices featuring the BG and MTL for LGI1‐AE classification. These findings align with prior studies reporting consistent metabolic abnormalities in the MTL and BG of LGI1‐AE patients [40, 41], validating the model's capacity to detect subtype‐specific PET signatures. MTL hypermetabolism is reported as the dominant PET feature in GABAB‐AE [42, 43], with some patients exhibiting BG involvement [43]. Consistently, our model prioritized MTL and BG regions when analyzing PET slices, aligning with literature‐derived metabolic signatures. The GAD65 enzyme is vital for the synthesis of GABA, a key inhibitory neurotransmitter, and elevated anti‐GAD65 autoantibodies serve as markers of autoimmune neurological disorders [44]. Brain pathology in GAD65‐AE primarily localizes to limbic areas, with ^18^F‐FDG PET typically demonstrating MTL hypometabolism [36, 45]. Our model recapitulated this pattern, detecting metabolic abnormalities concentrated in the MTL—validating its utility for subtype‐specific PET classification. Together, these findings highlight the m‐MIL model's capacity to discriminate AE subtypes based on regional metabolic profiles, despite its reliance on lesion‐containing slices rather than focal lesions.

The primary limitation of this study is the relatively small sample size, attributed to the low incidence of AE. Further research is essential to assess the method's generalizability to larger cohorts and clarify its clinical feasibility. Additionally, this study analyzed lesion‐containing image slices rather than specific lesion locations. Exploring precise lesion localization may offer added value in future investigations.

## 5 Conclusion

Here, an attention mechanism was used to weight instances in order to aid efforts to focus on those instances of the greatest importance for distinguishing AE patients from controls and among AE subtypes. The developed attention‐based MIL model outperformed traditional algorithms in multiple classification tasks, reliably identifying metabolic abnormalities in AE patients' PET images. This innovative approach shows significant promise as a diagnostic and research tool for AE, offering new avenues for precision disease management.

## Author Contributions

Q.W. and Y.S. concepted, designed, and supervised the study. Y.S., and L.A. acquired the data. Y.S., J.L., Q.K., C.D., B.W., X.H., M.C., Y.J., L.Y., R.L., L.A., X.Y., Y.C., and R.S. analyzed and interpreted the data, provided statistical analysis, had full access to all of the data in the study, and are responsible for the integrity of the data and the accuracy of the data analysis. Y.S. and R.S. drafted the manuscript; Q.W. critically revised the manuscript for important intellectual content. All authors read and approved the final manuscript.

## Ethics Statement

The local ethics committees of each participating hospital reviewed and approved this study. This study utilized respectively collected data and was approved by the Institutional Review Board of Capital Medical University, Beijing Tiantan Hospital (Beijing, China) (KY2023‐079‐01), Clinical Medical College, Jining Medical University (Jining, China) (EC‐20230322‐1001), Hebei Yanda Hospital (Sanhe, China) (2024‐06‐001), and People's Hospital of Henan (Zhengzhou, China) (20240711‐1001).

## Consent

All participants or their caregivers provided written informed consent for the publication.

## Conflicts of Interest

The authors declare no conflicts of interest.

## Data Availability

The data that support the findings of this study are available from the corresponding author upon reasonable request.
